# Towards accurate imputation of quantitative genetic interactions

**DOI:** 10.1186/gb-2009-10-12-r140

**Published:** 2009-12-10

**Authors:** Igor Ulitsky, Nevan J Krogan, Ron Shamir

**Affiliations:** 1 Blavatnik School of Computer Science, Tel Aviv University, Tel Aviv 69978, Israel; 2 Department of Cellular and Molecular Pharmacology, University of California San Francisco, San Francisco, CA 94158, USA; 3 Current address: Whitehead Institute for Biomedical Research, 9 Cambridge Center, Cambridge, MA 02142, USA

## Abstract

Recent technological breakthroughs have enabled high-throughput quantitative measurements of hundreds of thousands of genetic interactions among hundreds of genes in *Saccharomyces cerevisiae*. However, these assays often fail to measure the genetic interactions among up to 40% of the studied gene pairs. Here we present a novel method, which combines genetic interaction data together with diverse genomic data, to quantitatively impute these missing interactions. We also present data on almost 190,000 novel interactions.

## Abstract 2

A new method for calculating quantitative genetic interactions allows for the inference of 190,000 new genetic interactions in *Saccharomyces cerevisae*.

## Background

Understanding the interactions between genes and proteins is essential for elucidating their function. Genetic interactions (GIs) describe the phenotype of a double knock-out in comparison to the phenotypes of single mutants, and they can be crudely classified into positive (alleviating), neutral, and negative (aggravating) interactions [1,2]. In a negative GI, the fitness (typically estimated by growth rate) of the double-mutant is lower than expected based on the fitness of single mutants. The most extreme example of a negative interaction is synthetic lethality, in which the joint deletion of two nonessential genes leads to a lethal phenotype. In a positive GI, on the other hand, the double mutant is healthier than expected. The expected fitness is usually defined as the product of the fitnesses of the single mutants [1,3,4].

In a genome of over 6,000 genes, such as that of *Saccharomyces cerevisiae*, there are some 18 million gene pairs, making the mapping of the complete genetic interactome a formidable challenge. Towards this goal, several techniques for high-throughput GI profiling have been developed. For example, two approaches, systematic genetic analysis (SGA) [5,6] and dSLAM (heterozygote diploid-based synthetic lethality analysis with microarrays) [7,8], have made it possible to screen for negative GIs, namely synthetic sick or synthetic lethal interactions, between a query gene and the collection of all nonessential genes. The recent introduction of E-MAP (epistatic miniarray profile) technology, which is an adaptation of SGA [9-12], has made it possible to quantitatively measure both positive and negative GIs among several hundreds of genes [9-11]. The largest published E-MAP to date [10] covers GIs between 743 *S. cerevisiae *genes involved in various aspects of chromosome biology. The use of quantitative GIs was shown to significantly improve gene function prediction [10].

Using the E-MAP technology, hundreds of thousands of GIs have been measured in *S. cerevisiae*. It is therefore appealing to use these data along with other genomic information to predict additional GIs. Wong *et al*. [13] pioneered the prediction of GIs in *S. cerevisiae*, using probabilistic decision trees and diverse genomic data, including mRNA expression, functional annotations, subcellular localization, deletion phenotypes and physical interactions. These authors also introduced '2-hop features' for capturing the relationship between a gene pair and a third gene. For example, if protein A physically interacts with protein C, and gene B is synthetic lethal with gene C, then the gene pair A-B possesses the characteristic '2-hop physical-synthetic lethal', which was shown to increase the likelihood of a synthetic lethal interaction between A and B. Assessment of the performance on SGA-tested gene pairs revealed sensitivity of 80% at a false positive rate of 18%. The 2-hop features were shown to be the most effective features for prediction of GIs, and omission of other individual features did not significantly hurt the performance. This result suggested that most negative GIs occur between pairs of compensating physical pathways. This phenomenon has since been extensively studied [14-18]. Zhong and Sternberg [19] used similar ideas and combined diverse genomic information from three species to predict synthetic lethal interactions in *Caenorhabditis elegans *using a logistic regression classifier. Paladugu *et al*. [20] focused on features based on protein-protein interaction (PPI) networks, such as node degree, centrality, and clustering coefficient. Using a support vector machine classifier, they showed that using PPI network information together with 2-hop features is sufficient for predicting synthetic lethality at about 90% accuracy.

Recently, Qi *et al*. [21] devised the first GI prediction scheme based solely on GI data. Observing that genetically interacting gene pairs are connected by many odd-length paths in the GI network, they developed a graph diffusion kernel that successfully predicts novel GIs. Combining this kernel with kernels based on other genomic data had little effect on prediction accuracy, leading them to conclude that most of the information needed to predict new GIs can be found in the existing GI network. Another method for predicting negative GIs using random walks has been recently proposed by Chipman and Singh [22].

All available methods for predicting GIs were designed and tested on synthetic sick or synthetic lethal GIs obtained with the SGA method [5,6]. SGA differs from E-MAP in two key aspects. First, SGA screens are inherently asymmetrical, as a relatively small set of 'baits' are tested against a genome-wide collection of 'preys'. Using E-MAP, all pairwise interactions among a subset of the genes are tested. Second, E-MAP is quantitative and is capable of capturing both positive and negative GIs. Unfortunately, for technical reasons E-MAPs contain a large number of missing interactions. In the ChromBio E-MAP, for example, over 34% of the interactions were not measured. The fraction of interactions that are missing is higher for essential genes (46% on average), but is similar for genes with reduced fitness in rich media and for other non-essential genes (29% and 33%, respectively). It is logical to surmise that the vast number of interactions measured in the available E-MAPs can be used to predict the unmeasured GIs. The unique features of E-MAPs suggest that a dedicated approach to prediction of missing GIs in E-MAPs may be more powerful than previously suggested techniques for GI prediction. It is this possibility that we address here.

Most of the previous studies on GI prediction were based on a large variety of genomic information available for each gene in *S. cerevisiae*. An exception are the studies by Qi *et al*. [21] and Chipman and Singh [22], which showed that information about the GI network alone is sufficient for a relatively accurate qualitative prediction of negative GIs. Here we show that by integrating GI information across genes, it is possible to achieve quantitative prediction of both positive and negative GIs that significantly outperforms predictions made by other methods. Furthermore, this prediction can be improved by combining E-MAP-based information with other genomic data, although this improvement is relatively minor. We thus show that the measured gene pairs in the E-MAP are the best source of information for predicting the pairs that could not be measured.

**Figure 1:**
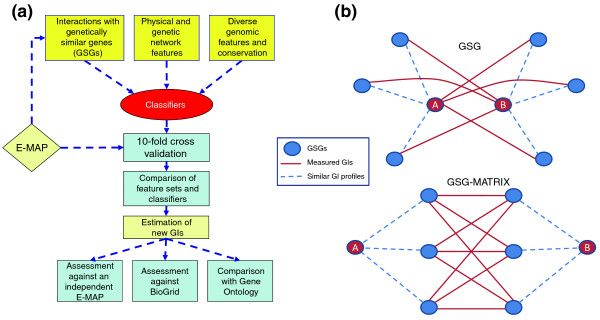
**Study outline and new features used**. **(a) **Study outline. Diverse features of gene pairs were computed and used to predict GIs using various classifiers. Performance was assessed by ten-fold cross-validation and the best combination of feature groups and classifier was selected. This combination was used to predict new GIs, which were subsequently tested against an independent E-MAP and negative interactions reported in the BioGrid database. In addition, we tested the correlation between the new GIs with functional similarity based on Gene Ontology. **(b) **Illustration of the GSG and GSG-MATRIX features. We were interested in predicting the GI between genes A and B. GSG features capture measured GIs between A and genes similar to B and vice versa. GSG-MATRIX features capture measured GIs between genes similar to A and genes similar to B.

The outline of our study was as follows (Figure 1a). We experimented with a variety of genomic features describing gene pairs, such as the existence of a physical interaction or co-expression, that were used as input to several popular classifiers. Some of the features are akin to previous ones and some are novel. We tested several popular methods that use the features to classify unknown GIs. To evaluate the quality of the combination of a particular feature set and a classifier, we applied a cross-validation procedure in which a fraction of the measurements were hidden and the ability of the classifier to recover them was assessed. The best performing algorithm was linear regression using all the possible features. Using data from three E-MAPs, we predicted 189,985 GIs among 144,498 pairs (some gene pairs appear in more than one E-MAP; see below). For a qualitative prediction of the GI type, we found that the best method was logistic regression using all features: it enabled us to identify over 40% of the missing strong positive and strong negative GIs in the ChromBio E-MAP by testing only 10% of the gene pairs, achieving four-fold improvement over random testing of pairs. The accuracy of our qualitative and quantitative predictions was further assessed with GI information from two additional independent sources.

We demonstrate the utility of the imputed E-MAP values for two tasks: to improve the ability to detect functionally similar genes using either predicted interactions or correlations of imputed GI profiles; and to more fully inspect the landscape of GIs among co-complexed genes. Finally, we address three scenarios that give rise to missing values in E-MAPs and discuss the ability of our method to predict a substantial number of new interactions through a combination of E-MAPs.

## Results and Discussion

### Construction of gene-pair feature sets

We analyzed three publicly available E-MAP datasets: the ChromBio dataset [10] containing GIs among 743 genes involved in chromosome biology; the endoplasmic reticulum (ER) dataset [9] containing GIs among 423 genes involved in the early secretory pathway; and the RNA dataset [12] containing GIs among 552 genes involved in RNA processing. We report mainly on the results from the ChromBio E-MAP, since it is the largest. Results on the two other E-MAPs are presented in Additional file 1. We computed a large number of features for each pair of genes in the E-MAP (Table 1; see Materials and methods for a description of how each feature was computed). These features can be crudely divided into four groups.

**Table 1:** Features used in this study

Feature group	Characteristic	Number of features	Data source	Previous use for GI prediction
NETWORK	Physical interaction	1	BioGrid [28]	[13,20]
	Shortest physical path	1	BioGrid [28]	[13,20]
	Mutual clustering coefficient	1	BioGrid [28]	[13,20]
	Network degree	6	BioGrid [28] and E-MAP	[20]
	2-hop	6	BioGrid [28] and the E-MAP	[13]
				
GENOMIC	Sequence similarity (BLAST E-value)	1	[45]	[13]
	Occurrence in a specific protein complex	32	MIPS [42]	-
	Co-occurrence in any protein complex	1	MIPS [42]	[13]
	Deletion phenotype	53	MIPS [42]	-
	A common deletion phenotype	1	MIPS [42]	[13]
	Correlation of quantitative phenotype profiles	1	[44]	-
	Gene Ontology semantic similarity	3	GO [58]	[13]
	Subcellular localization	17	[46]	-
	A common subcellular localization	1	[46]	[13]
	S-score in *S. pombe*	1	[11]	-
	mRNA expression (correlation)	7	[47-53]	[13]
				
GSG	S-score between A and genes similar to B (or vice versa)	10	E-MAP	-
				
GSG-MATRIX	S-scores among genes similar to A and to genes similar to B	25	E-MAP	-

The features are computed for every pair (A-B) of genes. Numbers of certain features depend on the E-MAP and are reported for the ChromBio E-MAP. SL, synthetic lethal; SS, synthetic sick.

The first two groups contain features that were used in previous studies [13,20]: the NETWORK group, which includes features based on the physical and GI networks, and the GENOMIC group, which includes features based on various genomic characteristics. Unlike previous studies, we defined separate individual features for each protein complex, phenotype and localization, whereas others used a single feature, encoding whether the gene pair shares any complex, phenotype or localization. This change stemmed from observations that some complexes tend to take part in a large number of GIs [15,16].

The third and the fourth groups constitute the main innovation in our feature set compared to previous works - the use of information on genetically similar genes (GSGs; Figure 1b; Materials and methods). The GI profile of a gene is a vector representing the scores of its GIs with other genes that took part in the GI screen. Previous studies have shown that similarity of GI profiles is a powerful indicator of functional similarity between genes [9,10,18,23]. Following this reasoning, we hypothesized that when predicting the GI between genes A and B, it would be useful to detect genes with GI profiles similar to those of A and B and to check the GIs among them (Figure 1b). We call a set of genes GSGs of gene A if their GI profiles are the most similar to those of A among all the genes in the E-MAP. The third group is called the GSG feature set. When we wish to predict the GI between genes A and B, it contains the GI scores (which, following [24], we call S-scores) between A and the GSGs of B and vice versa (see Materials and methods).

Recent studies have shown that many GIs occur between pairs of functional modules [15-18]. If A and B belong to distinct functional modules, it is reasonable that the S-scores between other members of the same module will be indicative of the S-score between A and B. This is the rationale behind the fourth group, called GSG-MATRIX, which contains S-scores between GSGs of A and GSGs of B (see Materials and methods). For the ChromBio E-MAP we used 15 NETWORK, 117 GENOMIC, 10 GSG and 25 GSG-MATRIX features (167 features in total).

### Comparison of feature sets and classifiers for prediction of quantitative GIs

**Figure 2:**
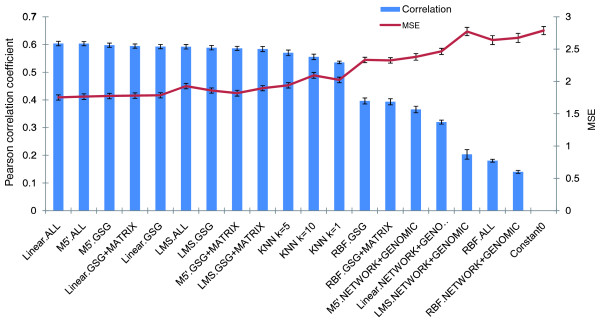
**Accuracy of prediction of quantitative GIs**. The combinations of classifier and feature sets are sorted in decreasing order of correlation of predicted values with the hidden S-scores. KNN, *k*-nearest-neighbor; Linear, linear regression; LMS, least median squared linear regression; MSE, mean square error; RBF, radial basis function classifier. Error bars indicate one standard deviation.

We distinguish between two tasks of GI prediction: estimation of the quantitative S-scores between genes; and discrete classification of GIs as positive, negative or neutral. The former task requires a classifier capable of predicting a numeric value for a gene pair, sometimes referred to as a regressor. We compared four regressors (Table 2). The performance was tested using: GSG features only; GSG and GSG-MATRIX features (called GSG+MATRIX); NETWORK and GENOMIC features (both used in previous studies); and all four groups of features. We determined the utility of using complex machine learning algorithms by testing *k*-nearest-neighbors-like classifiers, which estimated the GI between A and B as the average of their GSG features. Finally, we tested a 'blind' classifier that predicts all GIs to be completely neutral (that is, with S-score 0). Using ten-fold cross-validation, we computed the correlation between the predicted and the actual S-scores, and the mean square error of each combination of a classifier and a feature set. The results are presented in Figure 2. We obtained the best performance when using linear regression together with all the features, with similar results obtained using the more computationally intensive M5' (a decision tree with regression models at its leaves [25]). Using M5' or linear regression with GSG+MATRIX features yielded near-optimal results. Overall, these features showed great advantage over those in the NETWORK or GENOMIC groups. The GSGs - that is, the genes with the most similar GI profile to the tested genes - were ranked according to the similarity values. In the linear regression model for GSG features, as expected, features corresponding to GSGs of higher rank were given a higher weight (Figure S1 in Additional file 1). The results show that this weighting gives a clear advantage over using unweighted *k*-nearest-neighbors classifiers (Figure 2). The utility of the GSG features did not depend heavily of the number of GSGs used, as GSGs of order >5 consistently attained low weights (Figure S2 in Additional file 1). We got very similar results with the same analysis using the ER and the RNA E-MAPs (Figure S3 in Additional file 1). Imputed versions of all three E-MAPs obtained using linear regression with all features are available in Additional file 2. Due to its superiority over other methods, we used linear regression with all the features in all further experiments (unless indicated otherwise).

**Table 2:** Classifiers used in this study

Task	Classifier	Reference
Quantitative GI prediction	Linear regression	[59]
	M5'	[60]
	Least median squared linear regression	[61]
	Gaussian radial basis function network	[62]
	*k *nearest neighbors	[59]
		
GI class prediction	Naïve Bayes	[59]
	Random Forest	[63]
	J48 decision tree	[59]
	Logistic regression	[64]
	Discretized linear regression	See Materials and methods
	Diffusion kernel	[21]

### Comparison of feature sets and classifiers for prediction of GI class

We also tested different combinations of feature sets and classifiers for qualitative prediction of GIs. The GIs in the training set were assigned to be positive, negative or neutral (see Materials and methods), and the classifiers were trained to predict the three classes. We compared five classifiers (Table 2), including those used in previous GI prediction studies [13,19]. We also compared our approach to the diffusion kernel method recently proposed by Qi *et al*. [21] (using the original implementation provided by the authors, which we applied to the same dataset; see Materials and methods). We used the *G*^- ^diffusion kernel (based on the number of odd-length paths between the two genes) for prediction of negative interactions, and the *G*^+ ^kernel (based on the number of even-length paths) for prediction of positive interactions (see Materials and methods). An implementation of the random walk method of Chipman and Singh [22] was not available for comparison. Classifier performance was evaluated separately for prediction of positive and negative interactions, using two criteria. First, as in previous studies, we computed the area under the curve (AUC) score; this is the area under the receiver operating characteristic (ROC) curve, which plots the fraction of true positives as a function of the false positive rate, as the prediction threshold varies [26]. Although widely used, the AUC criterion is not very informative in our case because the dataset is skewed: there are many more negative than positive examples (the ratio between negative, positive and neutral interactions is approximately 6:3:91 in the ChromBio E-MAP and 3:2:95 in the ER and RNA E-MAPs). In the case of GI prediction, it is especially important that there be a sufficient fraction of true positives among the best-ranked predictions that could potentially be experimentally tested. One way to quantify this is to look at the precision-recall curve, which plots the fraction of the predictions that are correct as a function of the true positive rate (the fraction of true pairs that were predicted correctly) [27]. The area under the precision-recall curve (AUPR) provides a better quantitative assessment of the performance when the dataset is skewed. A method with perfect classification accuracy has an AUC of 1 and an AUPR of 1, while a random classifier would have an AUC of 0.5 and (for data with a low fraction of positive examples) an AUPR close to 0.

**Figure 3:**
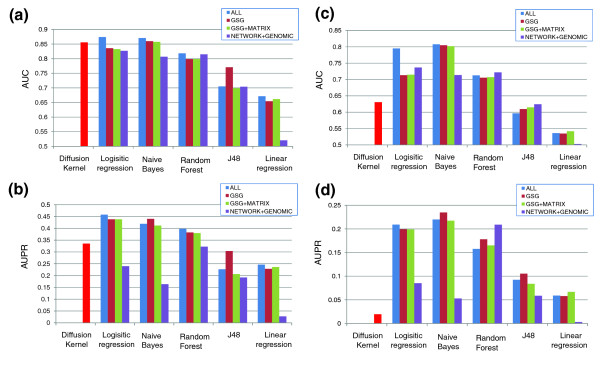
**Accuracy of qualitative GI prediction**. The histograms compare combinations of classifiers and feature sets when seeking a classification of gene pairs into positive, negative and neutral interactions. The combinations are compared in terms of the area under the ROC curve (AUC) and the area under the precision-recall curve (AUPR). **(a, b) **Predictions of negative interactions, measured by the AUC (a) and AUPR (b). **(c, d) **Predictions of positive interactions using AUC (c) and AUPR (d). The diffusion kernel method [21] uses only the topology of the GI network and does not exploit the other features.

The results are presented in Figure 3. The best performance was achieved using all the features with the logistic regression or Naïve Bayes classifiers. Using GSG or GSG+MATRIX features, it was possible to obtain near-optimal classification accuracy, and these features significantly outperformed classifiers using only network or genomic properties, which were used in previous studies. The G^- ^diffusion kernel was indeed very powerful in predicting negative interactions, especially given the amount of information it used (only the synthetic lethal interactions). However, the *G*^+ ^kernel performed rather poorly in predicting positive interactions. In general, the prediction of negative GIs appears to be easier than the prediction of positive GIs, since most methods fared much better on the former task. The higher difficulty of predicting positive interactions was manifested for a variety of S-score thresholds used to define those interactions, as the AUPR for prediction of positive interactions did not exceed 0.25 for any threshold (Figure S4 in Additional file 1).

**Figure 4:**
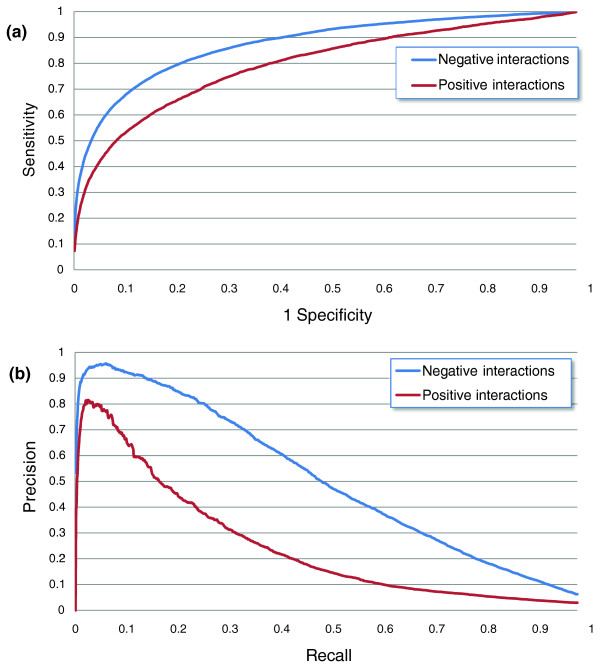
**Qualitative GI prediction using logistic regression and all the features**. Performance was evaluated by ten-fold cross-validation on the ChromBio E-MAP. **(a) **Receiver operating characteristic (ROC) plot. **(b) **Precision-recall plot.

Using logistic regression with all features, we find that we can obtain a recall of 40% of negative interactions by testing roughly 4.2% of the interactions at a precision of 61% (Figure 4), a significant improvement over the 45% precision for the same recall reported in [20]. Prediction of positive interactions is significantly more difficult, and recall of 40% requires testing 5.3% of the interactions at 20% precision. Thus, testing about 10% of the top predictions (9,300 gene pairs overall in the ChromBio E-MAP) is enough to discover over 40% of the significant positive and negative GIs that are missing.

### Accurate imputation of negative GIs not measured in the E-MAP

**Figure 5:**
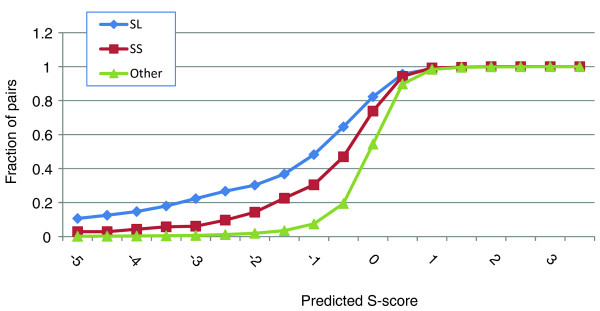
**Predicted S-scores for different groups of gene pairs**. The groups are categorized as synthetic lethal (SL) or synthetic sick (SS) according to the BioGrid database. The gene pairs in these groups were all missing in the ChromBio EMAP. 'Other' indicates all other pairs in BioGrid. The cumulative density function is shown for each group of gene pairs.

As an additional test for the accuracy of our method in prediction of negative GIs, we looked for pairs of genes from the ChromBio set with reported GIs that were not measured in the ChromBio E-MAP. We found 376 (279) synthetic lethal (sick) pairs with these properties in the BioGrid database [28]. The distribution of S-scores predicted for these pairs using linear regression and GSG+MATRIX features is shown in Figure 5. Note that here all the GI information originated from the E-MAP, and no information from BioGrid was used to construct the GSG+MATRIX features. Gene pairs marked as synthetic lethal in BioGrid had lower predicted S-scores (average = -1.81) than those marked as synthetic sick (average = -0.82, *t*-test *P*-value = 4.7 × 10^-9^) and than all other gene pairs in the ChromBio E-MAP (average = -0.14, *t*-test *P*-value <10^-200^). We also tested a discrete classifier, the Naïve Bayes classifier, and found that 174 (47.4%) of the gene pairs marked as synthetic lethal in BioGrid were predicted to be negative by our method. This fraction is likely to be an underestimate for the sensitivity of our method, as GIs in BioGrid were obtained in a variety of strains and conditions that were not necessarily the same as those used for the ChromBio E-MAP. Note that it is not possible to use BioGrid to estimate the specificity of our method, as it aggregates only successful negative GI detections from many high- and low-throughput studies, and it is not known which gene pairs were actually tested unsuccessfully in each study. Unfortunately, we could not use BioGrid to validate our positive interaction prediction accuracy: BioGrid contained only 76 pairs with unambiguous positive interactions that were not measured in the E-MAP, and this number was too small for evaluating our prediction accuracy (results not shown).

### Validation of quantitative predictions of GIs

While the comparison with BioGrid shows that our method is capable of predicting strong negative GIs, our main goals are to predict positive GIs and to make quantitative predictions. To test our ability to accomplish these goals, we used the RNA E-MAP, which shares 127 genes with the ChromBio E-MAP. Among these genes, we found 779 gene pairs for which GIs were measured only in the RNA E-MAP. These pairs could be effectively used as an independent test of our ability to predict quantitative GIs. When we imputed the missing values in the ChromBio E-MAP using linear regression with all the features, the correlation between the predicted values and the S-scores in the RNA E-MAP was 0.452 (Pearson correlation *P*-value = 2.2 × 10^-16^). While highly significant, this correlation is lower than the 0.604 we recorded in our cross-validation experiments using only the ChromBio E-MAP. A likely partial explanation for this is the E-MAP-specific normalization, which uses data from other genes in the same E-MAP to compute S-scores based on raw colony size measurements [24]. Similar to the results of the cross-validation experiments, the accuracy of the prediction of negative interactions was higher than that of positive interactions (52.5% versus 37.5%).

### Individual features most useful for prediction of GI type

In order to assess the features most useful for prediction of GIs, we ranked the features based on the absolute value of their correlation with the S-scores across the 182,057 gene pairs measured in the ChromBio E-MAP. The top 50 features are listed in Table 3 and the full list appears in Table S1 in Additional file 1. The comparison further emphasizes the high utility of the GSG features. Consistent with our findings in comparing different feature sets, the 29 top ranked features are all GSG and GSG-MATRIX features, and all 35 GSG+MATRIX features appear in the top 36 features. Not surprisingly, the three top features are the GIs between GSG_1_(A) and GSG_1_(B), A and GSG_1_(B), and B and GSG_1_(A) (GSG_i_(X) is the gene ranked *i *by GI profile similarity to *X*). As for other feature types, consistent with the results of [13], we found that among the features based on network and genomic information, the 2-hop features are very powerful, with five such features ranked in the top 50. We found '2-hop physical-synthetic lethal' the most useful 2-hop feature, consistent with the dominant role of GIs as bridging physical pathways [14-16]. Other high-ranking features include the average degrees of the gene pair in the synthetic lethal (ranked 29th), synthetic sick (37th) and physical (88th) networks. The physical and the genetic degree of a gene were shown to be correlated in *S. ceverisia*e [29]. The high ranks of these features indicate that genes already established to be involved in many genetic and physical interactions are likely to be involved in additional GIs. However, the presence of a physical interaction was only very weakly correlated with the measured S-scores (ranked 162nd), consistent with the observation that both strongly positive and strongly negative S-scores frequently correspond to physical interactions (see below). The highest ranking phenotype feature (ranked 46th) was 'slow growth', indicating that genes whose deletion limits the growth of the cell are likely to cause strong phenotypes when their deletion is accompanied by an additional knockout.

**Table 3:** The features with the highest correlation to measured S-scores

Number	Group	Feature	Correlation
1	GSG-MATRIX	GSG-MATRIX #1	0.505
2	GSG	GSG #1 for A	0.501
3	GSG	GSG #1 for B	0.491
4	GSG-MATRIX	GSG-MATRIX #2	0.489
5	GSG-MATRIX	GSG-MATRIX #3	0.419
6	GSG	GSG #2 for A	0.417
7	GSG	GSG #2 for B	0.412
8	GSG-MATRIX	GSG-MATRIX #4	0.403
9	GSG	GSG #3 for A	0.366
10	GSG-MATRIX	GSG-MATRIX #7	0.364
11	GSG	GSG #3 for B	0.358
12	GSG-MATRIX	GSG-MATRIX #8	0.341
13	GSG-MATRIX	GSG-MATRIX #6	0.329
14	GSG	GSG #4 for A	0.328
15	GSG-MATRIX	GSG-MATRIX #5	0.321
16	GSG-MATRIX	GSG-MATRIX #13	0.319
17	GSG	GSG #4 for B	0.310
18	GSG-MATRIX	GSG-MATRIX #9	0.294
19	GSG-MATRIX	GSG-MATRIX #14	0.293
20	GSG	GSG #5 for A	0.280
21	GSG	GSG #5 for B	0.280
22	GSG-MATRIX	GSG-MATRIX #12	0.271
23	GSG-MATRIX	GSG-MATRIX #10	0.270
24	GSG-MATRIX	GSG-MATRIX #21	0.270
25	GSG-MATRIX	GSG-MATRIX #11	0.264
26	GSG-MATRIX	GSG-MATRIX #15	0.257
27	GSG-MATRIX	GSG-MATRIX #22	0.248
28	GSG-MATRIX	GSG-MATRIX #16	0.242
29	GSG-MATRIX	GSG-MATRIX #20	0.235
30	NETWORK	SL degree (average of A and B)	-0.232
31	GSG-MATRIX	GSG-MATRIX #17	0.231
32	GSG-MATRIX	GSG-MATRIX #23	0.227
33	GSG-MATRIX	GSG-MATRIX #18	0.226
34	GSG-MATRIX	GSG-MATRIX #19	0.221
35	GSG-MATRIX	GSG-MATRIX #24	0.207
36	GSG-MATRIX	GSG-MATRIX #25	0.205
37	NETWORK	2-hop physical-SL	0.186
38	NETWORK	SS degree (average of A and B)	-0.164
39	GENOMIC	S-score in *S. pombe*	0.145
40	NETWORK	2-hop SL-SL	0.130
41	NETWORK	2-hop physical-SS	0.128
42	NETWORK	2-hop SS-SS	0.100
43	NETWORK	2-hop SL-SS	0.088
44	GENOMIC	GO cellular compartment similarity	-0.064
45	GENOMIC	Localization: Golgi	-0.047
46	GENOMIC	MIPS phenotype: Slow-growth	-0.045
47	GENOMIC	Quantitative phenotype correlation	-0.045
48	GENOMIC	Localization: microtubule	-0.039
49	GENOMIC	GO biological process similarity	-0.039
50	GENOMIC	Co-occurrence in any subcellular localization	0.038

The features are sorted by the absolute value of their correlation with measured S-scores. The features are computed between every pair A-B of genes. SL, synthetic lethal; SS, synthetic sick.

Our feature set contained separate features representing individual complexes, phenotypes or localizations. This information was summarized using a single feature in [13]. Thirteen individual complex features were ranked higher than the 'same MIPS complex' feature; 25 individual phenotype features were ranked higher than 'same MIPS phenotype'; and two localizations were ranked higher than 'same localization'. Hence, using individual features is indeed beneficial, as their information content frequently exceeds that of 'summary' features.

Finally, we compared the performance of each of the four groups of features separately with linear regression (Figure S5 in Additional file 1) and found that the performance of the GSG features alone was best, followed, in decreasing order, by GSG_MATRIX, NETWORK and GENOMIC groups. Note that this order is reversed to the number of features in each group, indicating that the quality of the features is much more important than their number.

### Gene pairs predicted to genetically interact are functionally related

**Figure 6:**
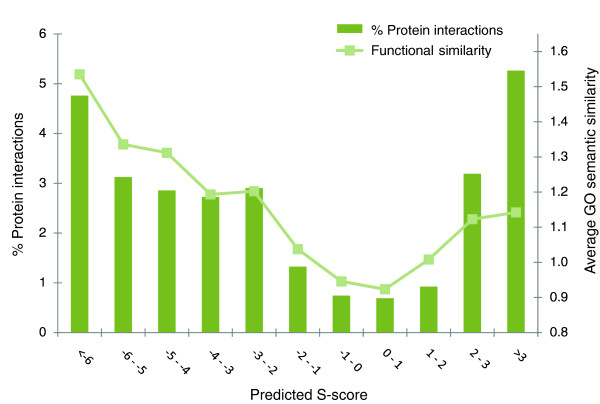
**Functional similarity for different ranges of predicted S-scores**. All the missing values in the ChromBio E-MAP were imputed using linear regression and GSG+MATRIX features and binned into 11 bins. The percent of protein interactions refers to the fraction of gene pairs that had a physical interaction between them reported in the BioGrid database. The average GO biological process semantic similarity was computed as described in [31].

Pairs of genes exhibiting positive or negative GIs were previously shown to be functionally related and likely to physically interact [10,12,18]. We therefore examined whether the GIs we predicted shared the same characteristics. To test this, we predicted the 93,596 missing values in the ChromBio E-MAP using linear regression and the GSG+MATRIX features. When predicted positive and negative GIs were tested separately, their absolute values were significantly correlated with an increasing functional similarity (*P *= 1.2 × 10^-7 ^and *P *< 2.2 × 10^-16 ^using Pearson correlation, for positive and negative interactions, respectively; functional similarity was measured using Gene Ontology (GO) semantic similarity [30], using the Resnik similarity measure [31]) and an increasing propensity for physical interactions (defined by the fraction of gene pairs reported to physically interact in the BioGrid database, *P *= 0.041 and *P *< 2.2 × 10^-16^; Figure 6).

### Imputation improves correspondence between genetic and functional similarity

**Figure 7:**
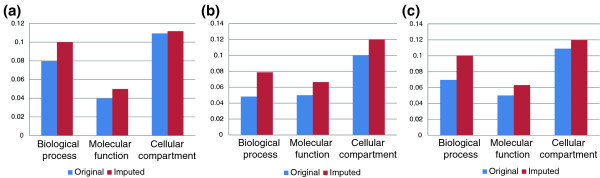
**The effect of missing value imputation on correlation with functional similarity**. The Pearson correlation between the similarity of GI profiles and the similarity of GO annotations (measured using GO semantic similarity [31]) was computed for the original and imputed data in each of the available E-MAPs. **(a) **Results on the ChromBio E-MAP. **(b) **Results on the ER E-MAP. **(c) **Results on the RNA E-MAP. To avoid bias, imputation did not use function-related features.

The results in the previous sections show that our method is capable of improving the accuracy of predicting GIs. One potential use of such prediction is to elucidate the functional relationship between two genes based on the prediction of the single GI between them. Another is through the use of GI profile similarity. We tested whether the imputation of missing values improves the ability to detect functionally similar genes using GI profile similarity. We used GO Resnik semantic similarity [31] to compute the functional similarity between every pair of genes in the E-MAP and then tested the correlation between functional similarity and GI profile similarity before and after the imputation. Imputation was performed using linear regression and GSG features (excluding features related to functional annotations in order to avoid bias). The results are presented in Figure 7. The imputation improves the correspondence between similarity of GI profiles and functional similarity by 27.6% on average. Interestingly, the difference was most profound in the ER E-MAP (Figure 7b), despite the fact that it has relatively few missing values (7.33% compared to 34% in the ChromBio E-MAP). We validated that the improvement occurs also when using Wang semantic similarity [32]. The results are shown in Figure S6 in Additional file 1. The imputation improves the correspondence between similarity of GI profiles and functional similarity in seven out of nine cases. The two exceptions occur with the 'molecular function' ontology and GI profile correlations in the ER and RNA E-MAPs.

### Predicted genetic interactions within protein complexes

We next analyzed the predicted landscape of GIs among genes belonging to the same protein complex. Bandyopadhyay *et al*. [17] studied the ChromBio E-MAP and found that many protein complexes are enriched with either positive or negative GIs, and that complexes enriched with negative interactions commonly carry out essential functions and thus are more likely to contain essential genes. However, several complexes, such as TFIID, TFIIF and Mediator, contained a very large number of missing values and therefore could not be reliably studied using the measured interactions. We performed imputation on the ChromBio E-MAP using linear regression and all the features, and inspected the fraction of positive and negative interactions among genes belonging to the same complex.

**Figure 8:**
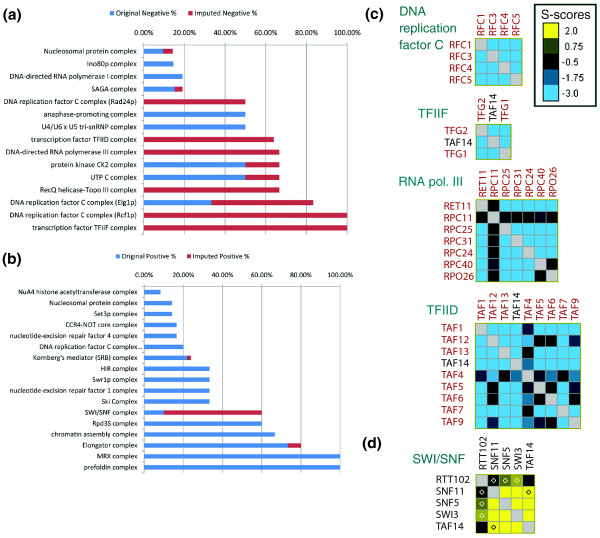
**Genetic interactions within protein complexes**. **(a, b) **Percent of gene pairs within complexes that have a negative GI between them. (a) Complexes with at least 20% negative interactions. (b) Complexes with at least 20% positive interactions. **(c, d) **Examples of representative protein complexes enriched with negative (c) or positive (d) GIs. For each complex the matrix presents the combined measured and predicted data. Measured GIs are marked by a yellow dot. No GIs were measured in the complexes in (c). Essential gene names are in red.

We selected all the complexes described in a recent yeast protein complex curation [33] that contained at least three genes in the ChromBio set. Of these complexes, 38 contained at least one positive or negative GI after imputation using linear regression and all the features; and 17 (15) were significantly enriched with positive (negative) GIs (false discovery rate < 0.05; see Materials and methods; Figure 8a, b). Bandyopadhyay *et al*. [17] identified 19 modules (corresponding to putative complexes or pathways) that were enriched for positive interactions and 9 enriched for negative ones. In contrast, we found that the number of complexes enriched with negative interactions is comparable to that of complexes enriched with positive interactions. This is probably because we were able to analyze additional complexes that are enriched with negative interactions (see below).

We were able to significantly increase the number of complexes that have predominantly negative interactions (Figure 8a). Four such complexes are shown in Figure 8c: DNA replication factor C, TFIIF, RNA polymerase III and TFIID. Among protein complexes enriched with positive GIs (Figure 8b), most of the interactions were measured ones, with the exception of the SWI/SNF complex, in which we predicted many positive GIs (Figure 8d). Consistent with the results of Bandyopadhyay *et al*. [17], in six out of the seven complexes in which the majority of the negative interactions were newly predicted ones, at least two-thirds of the complex members are essential. In contrast, none of the members of the SWI/SNF complex are essential.

We emphasize that gene essentiality was not part of the features used for GI prediction. Our results provide further evidence that complexes enriched with negative GIs are likely to carry out essential functions.

### The effect of missing values abundance and distribution on prediction accuracy

**Figure 9:**
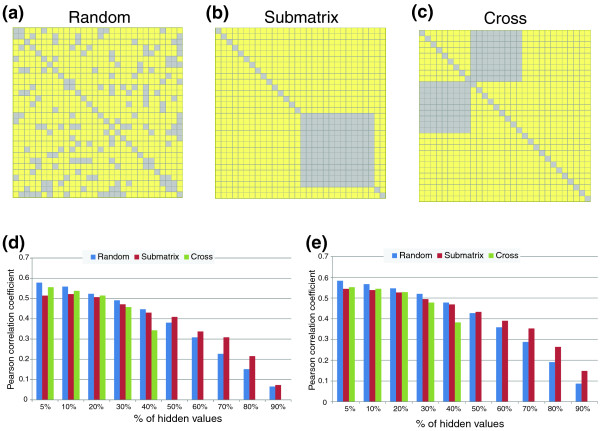
**The effect of missing values on the accuracy of quantitative GI prediction**. **(a-c) **The three scenarios producing missing values in E-MAP data. (a) In the 'Random' scenario, a random subset of gene pairs have hidden GIs. (b) in the 'Submatrix' scenario, a random subset of genes was selected and all the interactions between them were hidden. (c) In the 'Cross' scenario, two random disjoint subsets of genes were selected and all the interactions between them were hidden. In all three examples, 20% of the gene pairs were hidden. **(d, e) **Performance for different fractions of missing values in the three scenarios, using only the GSG+MATRIX features (d) or all the features (e). Performance was tested using the ER E-MAP, as it contained the least missing values. Imputation was performed by linear regression. The procedure was repeated 30 times. Performance was evaluated as the average Pearson correlation between the hidden and the predicted S-scores. Note that the 'Cross' scenario is not applicable for cases with ≥ 50% missing values.

Three scenarios can cause missing values in E-MAP experiments. In the first ('Random' model; Figure 9a), some gene pairs are not measured. In the second ('Submatrix' model; Figure 9b), all the interactions among a certain subset of genes (for example, essential genes) are missing. In the third scenario ('Cross' model; Figure 9c), all the interactions among two disjoint subsets of genes are missing. This last scenario arises, for example, if two E-MAPs that share a subset of their genes are combined into a new large E-MAP: all the interactions between genes that did not appear together in one original E-MAP are missing. The GSG+MATRIX features that we use rely heavily on 'borrowing' interaction data from similar genes. Hence, we compared the performance of a linear regression using these features alone or in combination with all other features in the three scenarios: for X = 5-90 we hid X% of the E-MAP measurements (a) randomly; (b) by first selecting a random subset of the genes and then hiding all the interactions between them; or (c) by first selecting two random disjoint sets of genes of equal size and then hiding all the interactions between them. Note that in the 'Cross' model it is not possible to hide more than 50% of the data. The ER E-MAP was used in this test, as it contained the fewest missing values.

The results are presented in Figure 9d, e. Our predictions were reasonably accurate (*r *> 0.4) when up to 50% of the E-MAP values were hidden for the 'Random' and 'Submatrix' models. For the 'Cross' model, performance already deteriorated when 40% of the data were removed. The performance was better than random (which results in correlation 0) even when up to 90% of the data were missing. As could be expected, when the fraction of hidden interactions was up to 40%, the prediction was more accurate in the 'Random' model than the 'Submatrix' model. Surprisingly, this trend was reversed when 50% or more of the data were hidden. A possible explanation for this phenomenon is that the number of common GI partners scales quadratically with the fraction of missing values for all the gene pairs in the first scenario, and scales linearly for some gene pairs in the second scenario (see Text S1 in Additional file 1 for a detailed explanation). With regard to the utility of our method for a combination of E-MAPs, we find that missing GIs can be predicted quite accurately (*r *> 0.4) when the two E-MAPs share ≥ 64% of their genes (which leads to ≤ 30% missing values). It is expected that as the percentage of missing GIs increases, the inclusion of NETWORK and GENOMIC features will be more helpful. Indeed, the difference between the performance using the GSG+MATRIX features only (Figure 9d) and using all the features (Figure 9e) was small (<10%) as long as ≤ 40% of the data were removed, but rose to above 20% when ≥ 70% of the data were removed.

## Conclusions

In this study we investigated prediction of quantitative GIs using data from E-MAP experiments. To the best of our knowledge, this is the first study attempting to address this problem. Our results suggest that such imputation is possible with about 60% accuracy by combining information from the available GI maps. Adding genomic data contributes only marginally to the prediction accuracy. This finding has important implications for the study of organisms other than *S. cerevisiae*, such as *Schizosaccharomyces pombe *for which two GI maps are already available [11,34], but other genomic data, such as PPIs, are still scarce. Our results show that imputation of missing values in future studies in such organisms will not be seriously affected by the lack of other genomic data.

The strength of the proposed approach is that it borrows information about GIs from related genes. This also underlines one of its limitations: it can only predict GIs among genes that have been studied genetically (that is, they appear in the same E-MAP). This limitation is shared by other methods utilizing only data about GIs [21], which are restricted to predicting GIs among genes that appear in the GI network.

To the best of our knowledge, this is also the first attempt to predict positive GIs. Our results show that the available approaches for predicting negative GIs perform poorly for prediction of positive interactions. While our method provides encouraging results in predicting such interactions, this task is evidently much more difficult than prediction of negative interactions. The accuracy of the best method to predict negative interactions is more than double that of the best method for prediction of positive interactions (0.45 versus 0.2 using the AUPR measure). One possible explanation for this difference in performance is that there are fewer positive interactions in the E-MAPs, and therefore less data points to properly train the classifiers. Another possibility is that the nature of these interactions is more complex than that of the negative GIs, making their prediction a more difficult task. Perhaps other, yet to be discovered features can predict these interactions with better accuracy.

The use of GI maps in yeast has already led to identification of novel complexes and gene functions, some of which were not recovered by other available methods [10,35-40]. It is thus expected that the use of such maps will increase, and large GI maps will be created for other biological systems (for example, mammalian cell lines) in the near future. As long as these maps remain prone to biological and technical noise, imputation of missing data will play a key role in their computational analysis.

## Materials and methods

### Data preprocessing

We used the S-scores reported in the original publications [9-12]. To avoid bias due to extreme S-scores, S-scores below -10 were set to -10 and S-scores above 10 were set to 10. When an open reading frame was represented by more than one deletion strain (for example, a knock-out strain and a strain with a DAmP allele [9]), the strain with the least missing values was chosen. When predicting the type of the GI, following [12], we defined a GI as negative if the S-score was below -2.5 and as positive if the S-score was above 2.

### Network and genomic feature sets

We now describe the features based on network properties and genomic information that we used. Previous studies that employed these features for GI prediction are listed in Table 1. We used three networks: PPI, and synthetic lethal and synthetic sick networks, all taken from BioGrid [28]. We added to the synthetic lethal network interactions between gene pairs from the analyzed E-MAP that had S-scores ≤ -2.5.

#### Physical interaction

Physical interaction is a binary feature indicating if the proteins interact in the physical network.

#### Network degree

Network degree is the number of neighbors in the PPI, synthetic lethal and synthetic sick networks recorded for each gene. Following [20] we used two features for each network and each gene pair with degrees *d*_1 _and *d*_2_: the average degree (d_1 _+ d_2_)/2 and the absolute difference between the degrees, |d_1 _- d_2_|.

#### Shortest physical path

The shortest physical path is the length of the shortest path between the proteins in the PPI network.

#### Mutual clustering coefficient

Mutual clustering coefficient was computed as described in [41] using the PPI network.

#### 2-hop

The 2-hop feature was computed as described in [13], using the physical, synthetic lethal and synthetic sick networks.

#### Protein complexes

Protein complexes were taken from the MIPS (Munich Information Center for Protein Sequences) database [42]. Only complexes in which at least three proteins appeared in the analyzed E-MAP were used. For each complex we added a ternary feature indicating how many of the proteins in the gene pair (0, 1 or 2) appeared as part of the complex. These features were called 'individual' as they refer to individual complexes. In addition we added a binary feature indicating whether the genes in the pair shared any protein complex. Using a newer collection of protein complexes [43] did not significantly affect the prediction performance (results not shown).

#### MIPS phenotypes

*S. cerevisiae *single deletion strain phenotypes (for example, sensitivity to DNA damaging agents) were obtained from MIPS [42]. Only phenotypes shared by at least three genes in the analyzed E-MAP were used. As for protein complexes, we added a ternary feature for each phenotype and a binary feature indicating whether the gene pair shared any phenotype.

#### Quantitative phenotype correlation

We used the quantitative measurements of single deletion phenotypes described in [44]. For each gene pair, we computed the Pearson correlation between the phenotypic profiles of the genes.

#### GO semantic similarity

Semantic similarity between the annotations of the two genes were computed using the method described in [31]. Similarity was computed separately for each part of the GO - 'biological process', 'molecular function' and 'cellular compartment'.

#### Protein sequence similarity

Translated open reading frames obtained from the Saccharomyces Genome Database [45] were BLASTed for quantifying the protein sequence similarity. The feature equals the -log(E-value) for the best local alignment found (if the best E-value was above 5 the feature was set to 0).

#### Subcellular localization

Subcellular localization for *S. cerevisiae *proteins was obtained from [46]. Only localizations shared by at least three genes in the analyzed E-MAP were used.

#### S-score in S. pombe

For each gene pair this feature contained the *S*-score between the orthologs of the genes in *S. pombe *(if available in the Pombe E-MAP [11]). Orthology assignments between *S. cerevisae *and *S. pombe *were taken from [11].

#### mRNA expression

We computed the Pearson correlation between the gene expression profiles of the genes in seven mRNA expression datasets [47-53]. Overall, 811 gene expression profiles were used.

### GSG and GSG-MATRIX features

For each gene A, we ordered all the other genes based on the similarity between their GI profile and the GI profile of A (using Euclidean distance as a measure of similarity). Gene B is called a GSG of A if it is among the genes most similar to A. A GSG of A is informative about B if the information about its GI with B is available (that is, it is neither missing nor hidden in the cross-validation experiments). The GSG feature set consists of 2 *k *features: for each gene pair A-B, it contains the S-scores between A and the *k *highest order GSGs of B that are also informative about A (called GSG #1 through GSG #k for A) and between B and the *k *top informative GSGs of A (called GSG #1 - k for B; Figure 1b; Figure S7 in Additional file 1). Note that since the gene pairs are not ordered, the *k *pairs of GSG features are symmetric (that is, GSG #1 for A and GSG #1 for B should be equally informative). Therefore, the small differences we observe between these feature pairs (Table S1 in Additional file 1) probably arise by pure chance. We used *k *= 5 throughout this study (see Figure S2 in Additional file 1 for the analysis of sensitivity to *k*).

The GSG-MATRIX feature set contains *k*^2 ^features representing the available S-scores between the top GSGs of A and the top GSGs of B (Figure S7 in Additional file 1). Due to missing values, typically there will be less than *k*^2 ^S-scores available between the top *k *GSG of A and the top *k *GSGs of B. We therefore used the following strategy. Denote by GSG_i_(A) the *i*-th GSG of A. In iteration *i *we added to the feature set the available S-scores between *GSG*_*i*_*(A) *and the *i *top GSGs of B and between *GSG*_*i*_*(B) *and the *i *top GSGs of A. Starting from *i *= 1, we increased *i *until *k*^2 ^features were constructed. In each iteration we iteratively increased *j *from 1 to *i *- 1 and added the features corresponding to the GIs between [GSG_*i*_(A), GSG_j_(B)] and between [GSG_*i*_(B), GSG_*j*_(A)]. The iteration was stopped once *k*^2 ^features were obtained. This way, we ensured that the feature set did not contain missing values and preferred features corresponding to genes more similar to A and B.

### Classifiers

We used the classifiers implemented in Weka [54]. A fast implementation of Random Forest was taken from [55]. All the classifiers were used with default parameters. For GI class prediction, the linear regression predicted values were treated as negative if the predicted score was ≤ -2.5 and positive if it was ≥ 2.

### Prediction of GIs using a diffusion kernel

We constructed a synthetic lethality network by combining interactions from BioGrid with interactions between genes whose S-score in the E-MAP was ≤ -2.5. The network was analyzed using supplementary MATLAB code from [21]. *G*^- ^kernel was used to predict negative GIs, and *G*^+ ^to predict positive GIs. Note that the *G*^+ ^was originally proposed for prediction of PPIs, but we found that it performed better than G^- ^for prediction of positive interactions (a task that was not addressed by Qi *et al*. [21]). We tested different values of the γ parameter between 1 and 40 and selected for each E-MAP the parameter value that obtained the best AUC.

### Cross-validation

The gene pairs with measured values in the analyzed E-MAP were divided into ten random groups. In each iteration (fold), nine of the groups were used to train the classifiers and their performance was evaluated using the tenth group. In order to enhance computational efficiency, only 30% of the ChromBio and 50% of the RNA E-MAP measured gene pairs were used as the training set in each fold (the subset used was chosen randomly).

### Enrichment of protein complexes with positive or negative interactions

We used the following procedure to evaluate if a protein complex *C *is enriched with positive (negative) interactions. Suppose *C *contains *k *positive interactions. We generated an unweighted graph *G*_*p *_in which the nodes are the genes in the E-MAP and an edge connects *v *and *u *in *G*_*p *_if there is a positive interaction between *u *and *v *in the E-MAP. We then generated 1,000 random degree preserving graphs using edge shuffling [56]. The empirical *P*-value of the enrichment of C with positive interactions was estimated as the fraction of these graphs that contained at least *k *edges between the nodes in *C*. An analogous procedure was used to estimate the significance of the enrichment of *C *with negative interactions. Complexes enriched with a false discovery rate < 0.05 were selected using the Benjamini-Hochberg procedure [57].

## Abbreviations

AUC: area under curve; AUPR: area under the precision-recall curve; E-MAP: epistatic miniarray profile; ER: endoplasmic reticulum; GI: genetic interaction; GO: Gene Ontology; GSG: genetically similar gene; PPI: protein-protein interaction; ROC: receiver operating characteristic; SGA: systematic genetic analysis.

## Authors' contributions

IU, NJK and RS conceived the study, and participated in its design. IU and RS developed the prediction method, analyzed the results and wrote the manuscript. All authors read and approved the final manuscript.

## Additional files

The following additional data are available with the online version of this paper: a Word document including Text S1, Figures S1-S7, and Table S1 (Additional file 1); S-scores in ChromBio, ER and RNA E-MAPs after imputation of missing values (Additional file 2).

## Supplementary Material

Additional file 1 Text S1: a proposed explanation for the results of the comparison of the random and the submatrix models. Figure S1: comparison of the linear regression coefficients of GSG features. Figure S2: performance and the number of GSG features. Figure S3: accuracy of prediction of quantitative GIs on the ER and RNA E-MAPs. Figure S4: accuracy of positive GI prediction as a function of positive GI definition. Figure S5: performance using each feature group separately. Figure S6: the effect of missing value imputation on correlation with functional similarity measured using the Wang method. Figure S7: construction of GSG and GSG-MATRIX features. Table S1: correlation between all the features used in this study and the measured S-scores. Click here for file

Additional file 2 S-scores in ChromBio, ER and RNA E-MAPs after imputation of missing values. Click here for file
