# Supplementary material for: Towards accurate imputation of quantitative genetic interactions
Source: Genome Biol. 2009 Dec 10;10(12):R140. doi: 10.1186/gb-2009-10-12-r140 (PMC2812947; doi:10.1186/gb-2009-10-12-r140)
Supplement: Additional file 1 — Text S1: a proposed explanation for the results of the comparison of the random and the submatrix models. Figure S1: comparison of the linear regression coefficients of GSG features. Figure S2: performance and the number of GSG features. Figure S3: accuracy of prediction of quantitative GIs on the ER and RNA E-MAPs. Figure S4: accuracy of positive GI prediction as a function of positive GI definition. Figure S5: performance using each feature group separately. Figure S6: the effect of missing value imputation on correlation with functional similarity measured using the Wang method. Figure S7: construction of GSG and GSG-MATRIX features. Table S1: correlation between all the features used in this study and the measured S-scores. [file gb-2009-10-12-r140-S1.doc]

**Text S1**

**A proposed explanation for the results of the comparison of the random and the submatrix models**

As presented in **Figure 9D**, when the fraction of hidden interactions was ≤40%, prediction was more accurate when the hidden gene pairs were chosen randomly. However, this trend was reversed when 50% or more of the data was hidden. This phenomenon can be explained as follows: If, for example, 70% of the GIs are randomly removed from each profile, then for any two genes A and B, the number of genes C for which the S-scores for both C-A and C-B are available drops to about 9% (0.3⋅0.3) of the E-MAP genes. In this case, the similarity of GI profiles becomes poor, and it is difficult to predict GIs. If, on the other hand, the 70% missing data occur in a specific submatrix, there are still ≈17% (1-√0.7) of the genes (denote them as X) that have a full GI profile. For the complement set (denoted X^C^), ≈83% of the interactions are hidden. In many cases, our method can find for a gene y∈X^C^, genes in X that share about 17% of the GI information with y and that can be used for a relatively faithful prediction of y's GIs.

**Supplementary Figures**

**Figure S1. Comparison of the linear regression coefficients of GSG features.** The marks represent different values of the *k* parameter (the number of GSGs used). Note that for every order *i* there are 2 GSG features (GSGi(*A*) and *GSG_i_(B*)) and their average is shown. The regression coefficients were obtained in a single fold of a 10-fold cross validation that used only the GSG features.

**Figure S2. Performance and the number of GSG features.** Using linear regression and the GSG features for different values of the *k* parameter, we measured the correlation between the predicted value and the measured S-score and the mean square error (MSE).

**Figure S3.** Accuracy of prediction of quantitative GIs on the ER )upper) and RNA (lower) E-MAPs. The combinations of classifier and feature sets are sorted in decreasing order of correlation of predicted values with the hidden S-scores. MSE: mean square error; Linear: linear regression; LMS: least median squared linear regression; RBF: radial basis function classifier.


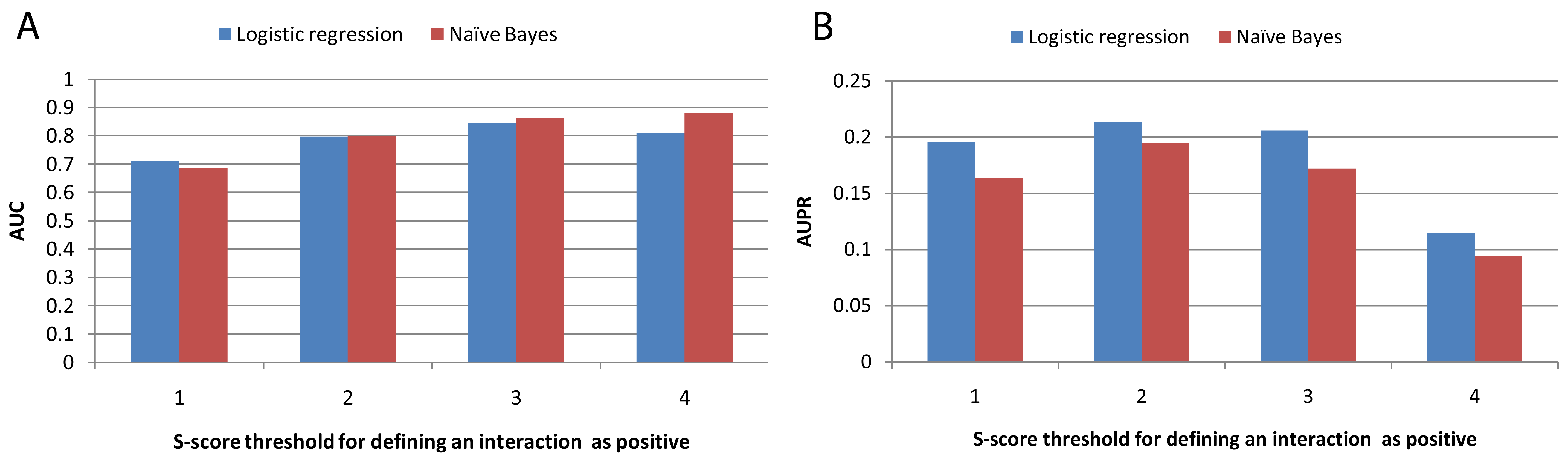


**Figure S4. Accuracy of positive GI prediction as a function of positive GI definition.** The histograms compare the two classifiers that gave the best performance using all the features when seeking a classification of gene pairs into positive and neutral interactions. The combinations are compared in terms of the area under the ROC curve (AUC, panel A) and the area under the Precision-Recall curve (AUPR, panel B). The X-axis shows the S-score threshold above which a gene pair was defined as having positive interaction.

**Figure S5. Performance using each feature group separately.** Performance was evaluated using the ChromBio E-MAP with 10-fold cross validation and linear regression. We measured the correlation between the predicted value and the measured S-score and the mean square error (MSE).


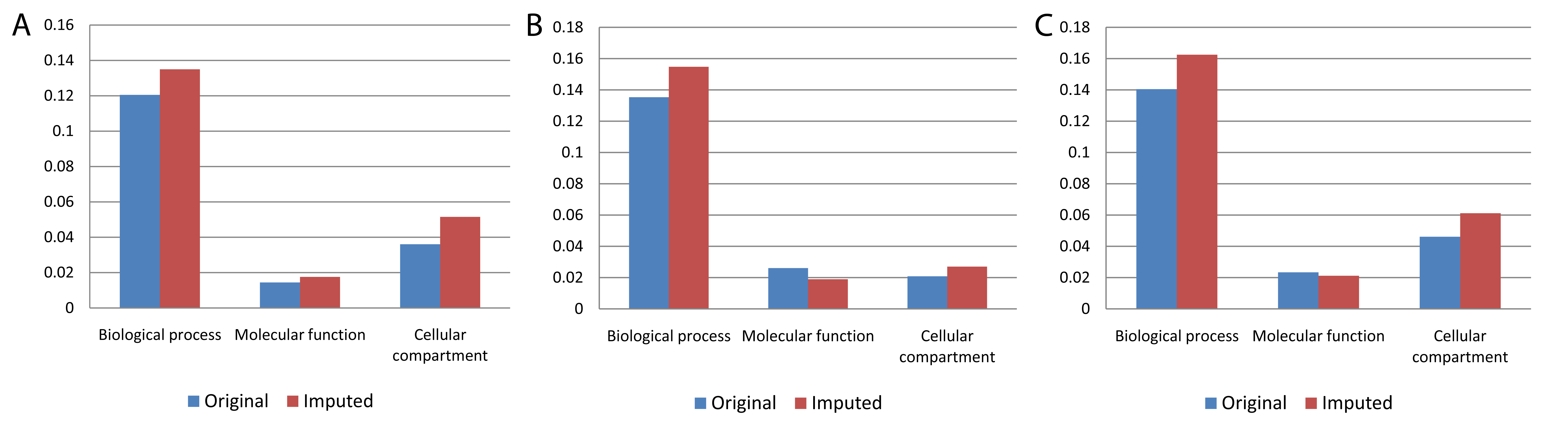


**Figure S6.** **The** **effect of missing value imputation on correlation with functional similarity measured using the Wang method.** The Pearson correlation between the similarity of GI profiles and the similarity of GO annotations (measured using GO semantic similarity as described in [32]) was computed for the original and imputed data in each of the available E-MAPs. (A) Results on the ChromBio E-MAP. (B) Results on the ER E-MAP. (C) Results on the RNA E-MAP. To avoid bias, imputation did not use function-related features.


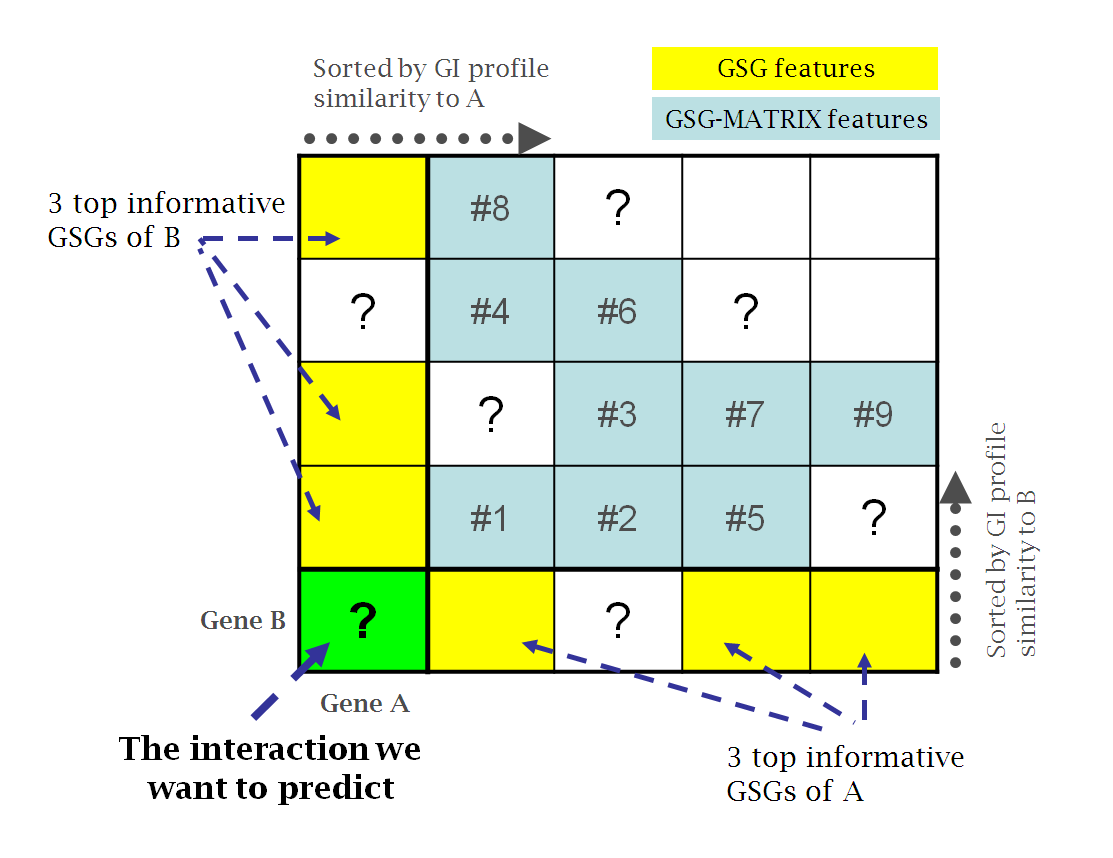
 **Figure S7. Construction of GSG and GSG-MATRIX features.** For every pair of genes A and B, the genes in the E-MAP are sorted based on the similarity of their GI profiles to the profiles of A and B. In the bottom row (respectively, left column), genes closer to the bottom-left corner have higher similarity to A (respectively B). The GSG features contain the S-scores between A and the *k* genes most similar to B for which GIs are available, and vice versa. Question marks represent missing values. In this example *k=*3*.* The GSG-MATRIX feature set contains *k^2^* GIs between the genes most similar to A and B. See Methods for the description of how this feature set is constructed.

| **Number** | **Group** | **Feature** | **Correlation** |
| --- | --- | --- | --- |
| 1 | GSG-MATRIX | GSG-MATRIX #1 | 0.505 |
| 2 | GSG | GSG #1 for A | 0.501 |
| 3 | GSG | GSG #1 for B | 0.491 |
| 4 | GSG-MATRIX | GSG-MATRIX #2 | 0.489 |
| 5 | GSG-MATRIX | GSG-MATRIX #3 | 0.419 |
| 6 | GSG | GSG #2 for A | 0.417 |
| 7 | GSG | GSG #2 for B | 0.412 |
| 8 | GSG-MATRIX | GSG-MATRIX #4 | 0.403 |
| 9 | GSG | GSG #3 for A | 0.366 |
| 10 | GSG-MATRIX | GSG-MATRIX #7 | 0.364 |
| 11 | GSG | GSG #3 for B | 0.358 |
| 12 | GSG-MATRIX | GSG-MATRIX #8 | 0.341 |
| 13 | GSG-MATRIX | GSG-MATRIX #6 | 0.329 |
| 14 | GSG | GSG #4 for A | 0.328 |
| 15 | GSG-MATRIX | GSG-MATRIX #5 | 0.321 |
| 16 | GSG-MATRIX | GSG-MATRIX #13 | 0.319 |
| 17 | GSG | GSG #4 for B | 0.310 |
| 18 | GSG-MATRIX | GSG-MATRIX #9 | 0.294 |
| 19 | GSG-MATRIX | GSG-MATRIX #14 | 0.293 |
| 20 | GSG | GSG #5 for A | 0.280 |
| 21 | GSG | GSG #5 for B | 0.280 |
| 22 | GSG-MATRIX | GSG-MATRIX #12 | 0.271 |
| 23 | GSG-MATRIX | GSG-MATRIX #10 | 0.270 |
| 24 | GSG-MATRIX | GSG-MATRIX #21 | 0.270 |
| 25 | GSG-MATRIX | GSG-MATRIX #11 | 0.264 |
| 26 | GSG-MATRIX | GSG-MATRIX #15 | 0.257 |
| 27 | GSG-MATRIX | GSG-MATRIX #22 | 0.248 |
| 28 | GSG-MATRIX | GSG-MATRIX #16 | 0.242 |
| 29 | GSG-MATRIX | GSG-MATRIX #20 | 0.235 |
| 30 | NETWORK | SL degree (average of A and B) | -0.232 |
| 31 | GSG-MATRIX | GSG-MATRIX #17 | 0.231 |
| 32 | GSG-MATRIX | GSG-MATRIX #23 | 0.227 |
| 33 | GSG-MATRIX | GSG-MATRIX #18 | 0.226 |
| 34 | GSG-MATRIX | GSG-MATRIX #19 | 0.221 |
| 35 | GSG-MATRIX | GSG-MATRIX #24 | 0.207 |
| 36 | GSG-MATRIX | GSG-MATRIX #25 | 0.205 |
| 37 | NETWORK | 2-hop Physical-SL | 0.186 |
| 38 | NETWORK | SS degree (average of A and B) | -0.164 |
| 39 | GENOMIC | S-score in *S. pombe* | 0.145 |
| 40 | NETWORK | 2-hop SL-SL | 0.130 |
| 41 | NETWORK | 2-hop Physical-SS | 0.128 |
| 42 | NETWORK | 2-hop SS-SS | 0.100 |
| 43 | NETWORK | 2-hop SL-SS | 0.088 |
| 44 | GENOMIC | GO cellular compartment similarity | -0.064 |
| 45 | GENOMIC | Localization: Golgi | -0.047 |
| 46 | GENOMIC | MIPS phenotype: Slow-growth | -0.045 |
| 47 | GENOMIC | Quantitative phenotype correlation | -0.045 |
| 48 | GENOMIC | Localization: microtubule | -0.039 |
| 49 | GENOMIC | GO biological process similarity | -0.039 |
| 50 | GENOMIC | Co-occurrence in any subcellular localization | 0.038 |
| 51 | GENOMIC | MIPS phenotype: Cold-sensitivity | -0.038 |
| 52 | GENOMIC | MIPS phenotype: Osmotic sensitivity | -0.036 |
| 53 | GENOMIC | Localization: nuclear periphery | -0.036 |
| 54 | GENOMIC | MIPS phenotype: Nuclear mutants | -0.034 |
| 55 | NETWORK | MIPS complex: Kinesin-related motorproteins | -0.033 |
| 56 | NETWORK | MIPS complex: Kornberg\s mediator (SRB) complex | -0.033 |
| 57 | GENOMIC | Localization: cytoplasm | 0.032 |
| 58 | GENOMIC | MIPS phenotype: Actin cytoskeleton mutants | -0.032 |
| 59 | NETWORK | MIPS complex: HDB complex | -0.032 |
| 60 | NETWORK | SL degree difference | -0.031 |
| 61 | GENOMIC | MIPS phenotype: Secretory mutants | -0.030 |
| 62 | GENOMIC | MIPS phenotype: Mating efficiency | -0.030 |
| 63 | GENOMIC | MIPS phenotype: Heat-sensitivity (ts) | -0.029 |
| 64 | GENOMIC | MIPS phenotype: 3-Aminotriazole sensitivity | -0.028 |
| 65 | NETWORK | Shortest PPI path | 0.027 |
| 66 | NETWORK | 2-hop Physical- Physical | 0.027 |
| 67 | NETWORK | MIPS complex: SPB associated proteins | -0.026 |
| 68 | GENOMIC | Expression correlation in Causton01 | -0.026 |
| 69 | GENOMIC | MIPS phenotype: other transcriptional mutants | -0.026 |
| 70 | GENOMIC | MIPS phenotype: other tubulin cytoskeleton mutants | -0.025 |
| 71 | GENOMIC | Localization: ER | -0.024 |
| 72 | NETWORK | MIPS complex: Gim complexes | -0.020 |
| 73 | NETWORK | SS degree difference | -0.020 |
| 74 | NETWORK | MIPS complex: SAGA complex | -0.019 |
| 75 | NETWORK | MIPS complex: Nucleosomal protein complex | 0.019 |
| 76 | GENOMIC | Expression correlation in Hughes00 | -0.018 |
| 77 | GENOMIC | MIPS phenotype: Sucrose fermentation (Snf;Ssn) | 0.018 |
| 78 | GENOMIC | MIPS phenotype: Flocculence | 0.018 |
| 79 | GENOMIC | Expression correlation in Gasch00 | -0.018 |
| 80 | GENOMIC | MIPS phenotype: other mating and sporulation defects | -0.017 |
| 81 | NETWORK | PPI network mutual clustering coefficient | -0.017 |
| 82 | GENOMIC | Localization: nucleolus | 0.017 |
| 83 | NETWORK | MIPS complex: NEF1 complex | 0.017 |
| 84 | GENOMIC | Expression correlation in ORourke04 | -0.017 |
| 85 | NETWORK | MIPS complex: MRE11/RAD50/XRS2 complex | -0.017 |
| 86 | GENOMIC | MIPS phenotype: Cycloheximide sensitivity | -0.017 |
| 87 | GENOMIC | Localization: nucleus | -0.016 |
| 88 | NETWORK | Physical degree average | -0.016 |
| 89 | GENOMIC | MIPS phenotype: other cell cycle defects | -0.015 |
| 90 | GENOMIC | MIPS phenotype: Inositol auxotrophy (Ino) | -0.015 |
| 91 | NETWORK | MIPS complex: TFIIF | -0.015 |
| 92 | NETWORK | MIPS complex: RNA polymerase II | -0.015 |
| 93 | GENOMIC | Protein sequence homology | -0.015 |
| 94 | GENOMIC | MIPS phenotype: UV light sensitivity | 0.015 |
| 95 | GENOMIC | MIPS phenotype: Hydroxyurea sensitivity | -0.015 |
| 96 | GENOMIC | GO molecular function similarity | -0.015 |
| 97 | GENOMIC | MIPS phenotype: Sporulation efficiency | -0.015 |
| 98 | NETWORK | MIPS complex: Synaptonemal complex (SC) | 0.015 |
| 99 | GENOMIC | Localization: actin | -0.014 |
| 100 | GENOMIC | MIPS phenotype: other carbon utilization defects | 0.014 |
| 101 | GENOMIC | Expression correlation in Spellman98 | -0.014 |
| 102 | NETWORK | MIPS complex: Tubulin-associated proteins | -0.014 |
| 103 | GENOMIC | MIPS phenotype: Galactose fermentation (Gal) | 0.013 |
| 104 | NETWORK | Co-occurrence in any MIPS complex | 0.013 |
| 105 | GENOMIC | Expression correlation in Gasch01 | -0.013 |
| 106 | NETWORK | Physical degree difference | 0.012 |
| 107 | GENOMIC | MIPS phenotype: other cell morphology mutants | -0.012 |
| 108 | NETWORK | MIPS complex: ADA complex | -0.012 |
| 109 | NETWORK | MIPS complex: Cdc28p complexes | 0.012 |
| 110 | GENOMIC | MIPS phenotype: BudLocalization | -0.012 |
| 111 | GENOMIC | Localization: spindle pole | -0.012 |
| 112 | NETWORK | MIPS complex: 19/22S regulator | -0.012 |
| 113 | GENOMIC | Same MIPS phenotype | 0.011 |
| 114 | GENOMIC | MIPS phenotype: Mutator phenotypes | 0.011 |
| 115 | NETWORK | MIPS complex: MSH2/MSH3 complex | 0.011 |
| 116 | GENOMIC | MIPS phenotype: Hygromycin B sensitivity | 0.011 |
| 117 | GENOMIC | MIPS phenotype: Silencing mutants | -0.010 |
| 118 | NETWORK | MIPS complex: TAFIIs | -0.010 |
| 119 | GENOMIC | MIPS phenotype: G1 arrest | -0.010 |
| 120 | NETWORK | MIPS complex: Srb10p complex | 0.010 |
| 121 | GENOMIC | Localization: punctate composite | 0.009 |
| 122 | GENOMIC | MIPS phenotype: Starvation sensitivity | -0.009 |
| 123 | GENOMIC | Localization: early Golgi | -0.009 |
| 124 | GENOMIC | MIPS phenotype: other vacuolar mutants | -0.009 |
| 125 | GENOMIC | MIPS phenotype: benomyl sensitivity | -0.008 |
| 126 | GENOMIC | MIPS phenotype: Respiratory deficiency | 0.008 |
| 127 | GENOMIC | MIPS phenotype: H2O2 sensitivity | 0.008 |
| 128 | GENOMIC | MIPS phenotype: other DNA repair mutants | 0.008 |
| 129 | GENOMIC | Localization: mitochondrion | 0.008 |
| 130 | GENOMIC | Localization: vacuole | -0.008 |
| 131 | GENOMIC | MIPS phenotype: other aminoacid analogs and other drugs | -0.007 |
| 132 | GENOMIC | MIPS phenotype: other or general oxidizing agents sensitivity | -0.007 |
| 133 | GENOMIC | MIPS phenotype: Elongated cell and bud morphologies | -0.007 |
| 134 | NETWORK | MIPS complex: other DNA repair complexes | -0.007 |
| 135 | GENOMIC | Localization: bud neck | 0.007 |
| 136 | NETWORK | MIPS complex: Replication factor C complex | -0.006 |
| 137 | NETWORK | MIPS complex: Replication complex | -0.006 |
| 138 | GENOMIC | MIPS phenotype: other cell wall mutants | -0.006 |
| 139 | GENOMIC | MIPS phenotype: Calcofluor white sensitivity | -0.006 |
| 140 | NETWORK | MIPS complex: TFIIH | -0.005 |
| 141 | NETWORK | MIPS complex: RNA polymerase III | -0.005 |
| 142 | GENOMIC | MIPS phenotype: Alkylating agents sensitivity | -0.004 |
| 143 | GENOMIC | MIPS phenotype: Pseudohyphae formation | 0.004 |
| 144 | NETWORK | MIPS complex: RNA polymerase I | 0.004 |
| 145 | GENOMIC | MIPS phenotype: nocodazole sensitivity | -0.004 |
| 146 | NETWORK | MIPS complex: NEF3 complex | 0.004 |
| 147 | NETWORK | MIPS complex: Chromatin assembly complex (CAC) | -0.004 |
| 148 | GENOMIC | Expression correlation in Robertson00 | -0.004 |
| 149 | GENOMIC | MIPS phenotype: Spindle mutants | -0.004 |
| 150 | NETWORK | MIPS complex: Casein kinase II | 0.004 |
| 151 | GENOMIC | Localization: cell periphery | -0.003 |
| 152 | GENOMIC | Localization: vacuolar membrane | -0.003 |
| 153 | GENOMIC | MIPS phenotype: Recombination mutants | 0.003 |
| 154 | GENOMIC | MIPS phenotype: Divalent cations and heavy metals sensitivity | -0.003 |
| 155 | GENOMIC | MIPS phenotype: Papulacandin B sensitivity | 0.003 |
| 156 | NETWORK | MIPS complex: RSC complex (Remodel the structure of chromatin) | 0.003 |
| 157 | GENOMIC | MIPS phenotype: other stress response defects | 0.003 |
| 158 | GENOMIC | MIPS phenotype: other DNA replication mutants | -0.003 |
| 159 | NETWORK | MIPS complex: SWI/SNF transcription activator complex | -0.002 |
| 160 | GENOMIC | MIPS phenotype: other carbohydrate and lipid biosynthesis defects | -0.002 |
| 161 | GENOMIC | Localization: ambiguous | 0.002 |
| 162 | NETWORK | Physical interaction | -0.002 |
| 163 | GENOMIC | MIPS phenotype: Heat shock sensitivity | -0.002 |
| 164 | GENOMIC | MIPS phenotype: G2/M arrest | -0.002 |
| 165 | GENOMIC | MIPS phenotype: Caffeine sensitivity | -0.001 |
| 166 | NETWORK | MIPS complex: SNF1 complex | 0.001 |
| 167 | NETWORK | MIPS complex: Ctf3 protein complex | -0.001 |

**Table S1. The correlation between all the features used in this study and the measured S-scores.** The features are color-coded based on the feature group and sorted by the absolute value of their correlation with measured S-scores. The features are computed between every pair A, B of genes. SL: synthetic lethal. SS: synthetic sick.
